# Reduction in oral mucositis severity using a topical vasoconstrictor: A case report of three bone marrow transplant patients

**DOI:** 10.15761/ICST.1000293

**Published:** 2018-11-29

**Authors:** Amanda Graul-Conroy, Margo Hoover-Regan, Kenneth B DeSantes, Paul M Sondel, Natalie S Callander, Walter L Longo, William E Fahl

**Affiliations:** 1 Department of Pediatrics, Hematology-Oncology Program, University of Wisconsin-Madison, USA; 2 Department of Medicine, Bone Marrow Transplant Program, University of Wisconsin-Madison, USA; 3 Wisconsin Institutes of Medical Research, University of Wisconsin-Madison, USA; 4 Department of Pediatrics, Hematology-Oncology Program, Baylor College of Medicine, USA; 5 Department of Medicine, Medical College of Wisconsin, USA

**Keywords:** cyclophosphamide, total body irradiation

## Abstract

**Background:**

Grade 3 oral mucositis (OM) is historically observed in >90% of bone marrow transplant patients who received the cyclophosphamide + total body irradiation (CY+TBI) conditioning regimen. It was previously shown that orotopically applied adrenergic vasoconstrictor prevented up to 100% of radiation-induced oral mucositis in two preclinical animal models.

**Methods:**

*A*drenergic vasoconstrictor (i.e., phenylephrine in an aqueous-alcohol NG11–1 formulation) was orotopically applied to three patients (ages 24–29) who received the CY+TBI conditioning regimen; they were compared to five matched controls who received no orotopical vasoconstrictor. All patients received the CY+TBI conditioning regimen for acute lymphoblastic leukemia within the University of Wisconsin Adult Bone Marrow Transplant Program. Over the seven-day Cy+TBI conditioning regimen, 20 min before each treatment, either radiation or chemotherapy, vasoconstrictor was applied topically to the oral cavity, and patients then received either 1.5 Gy whole-body radiation or IV cyclophosphamide.

**Results:**

OM severity was scored over a three-week period using: i) physican assessments, ii) daily photos of the oral cavity, iii) oral pain and oral function score sheets, and iv) recorded narcotic consumption. Both “Grade 3 OM” duration and “any OM” duration in vasoconstrictor-treated patients were substantially lower than for the five control patients. Though nasogastric tube or total parenteral nutrition were used in 3 out of 5 control patients, there was no use of these supportive care measures in the three vasoconstrictor-treated patients.

**Conclusion:**

Orotopically applied NG11–1 vasoconstrictor formulation substantially reduced the incidence and severity of “Grade 3” and “any” oral mucositis when compared to matched control patients, all of whom received the same CY+TBI conditioning regimen. The liquid orotopical formulation was easily tolerated by patients both in its ease of use and lack of side effects.

## Introduction

Oral mucositis (OM) occurs commonly in 40–80% of patients receiving high-dose chemotherapy conditioning regimens prior to hematopoietic stem cell transplants, in >90% of patients who receive the cyclophosphamide plus total body irradiation (CY+TBI) conditioning regimen [1–3], and in nearly 100% of irradiated head and neck cancer patients [4]. Common consequences of OM include pain, weight loss and dehydration from reduced oral intake, increased risk of infection, as well as prolonged hospitalization and increased healthcare costs [4,5], recently estimated at $17,000 (USD) per head and neck cancer patient [4]. The pathobiology of oral mucositis, which is initiated by chemotherapy or radiation insults to basal epithelial and vascular endothelial cells in the oral mucosa, has been described in detail by Sonis [6] and other groups [7]. Several strategies to manage OM have been pursued with limited success. Some of the strategies include palliation with mucosal barriers [8], topical and systemic antibiotics, analgesics and basic oral hygiene, and topical or systemic growth factors to enhance re-epithelialization of mucosa [9–11]. Palifermin was approved to reduce the severe OM seen in bone marrow transplant patients. Approximately 70% of ionizing radiation-induced death of cells, including the basal epithelial and vascular endothelial cells of oral mucosa, whose apoptosis is an early event in mucositis pathobiology, results from two rapid, sequential steps: i) radiation-induced reactive oxygen species (ROS) attack of DNA bases, and ii) molecular oxygen (O_2_) “fixation” of the ROS DNA base damage. Our earlier work showed complete suppression of radiation-induced oral mucositis [12] in two rodent models where mice or hamsters were treated once with topically-applied catecholamine vasoconstrictor minutes before irradiation. Though the ionizing radiation-induced ROS (mainly **·**OH) that attack DNA are largely formed by “splitting water molecules,” the molecular oxygen (O2) required to “fix” the DNA damage can be excluded from these cells by the topically-applied vasoconstrictor transiently excluding delivery of oxygen-bearing blood to the mucosal epithelium during irradiation. In this case, ROS-modified DNA nucleotides then “decay” (in milliseconds) back to their basal state. The efficacy of a topically applied vasoconstrictor can be visually observed by the “blanching” of mucosa or skin that occurs when oxygenated red blood (as well as any blood-borne chemotherapy it contains) is excluded from the ~1 mm deep layer of mucosa or skin which: i) lies above the submucosal/ subcutaneous vasculature, and ii) contains the mucosal, epidermal, or hair follicle “stem” cells, whose radiation- or chemotherapy-induced apoptosis initiates the oral mucositis, radiation dermatitis or alopecia side effects observed in cancer patients. The *transient hypoxia* of the surface oral or epidermal stem cells following topical application of vasoconstrictor, or the transient excluded delivery of systemic chemotherapy to oral/epidermal surface stem cells [13] is seen as a logical mechanism to confer chemo- and radio-protection to surface cells while not impeding killing of underlying leukemia [13] or solid tumor cells [14]. The topical vasoconstrictor approach provides both: i) flexibility in the duration of the protective effect and ii) absence of the discomfort associated with oral or scalp cryotherapy [15]. Topically applied adrenergic vasoconstrictor has shown 100% prevention of oral mucositis [12], alopecia [16] and radiation dermatitis [17] in animal models.

## Case description

### Patients 1, 2, 3

Based upon the orotopical vasoconstrictor-induced prevention of OM in animal models, an initial clinical study was approved by the University of Wisconsin Health Sciences Institutional Review Board to determine if an initial dose (16.3 mM phenylephrine in an aqueous-alcohol NG11–1 orotopical formulation) could suppress the incidence and severity of OM in CY+TBI patients. Three patients signed written informed consents for participation in the study and for publication of the clinical data as a case report. Institutional Review Board approval allowed study physicians to access pertinent information (including body weights, daily OM scores, narcotic consumption, use of total parenteral nutrition (TPN), etc.) from files of five patients who received the CY+TBI regimen at UW during the 12-month period preceding this study for comparison to the topical vasoconstrictor-treated patients.

### Treatment: bone marrow conditioning and topical vasoconstrictor

**Figure 1.**
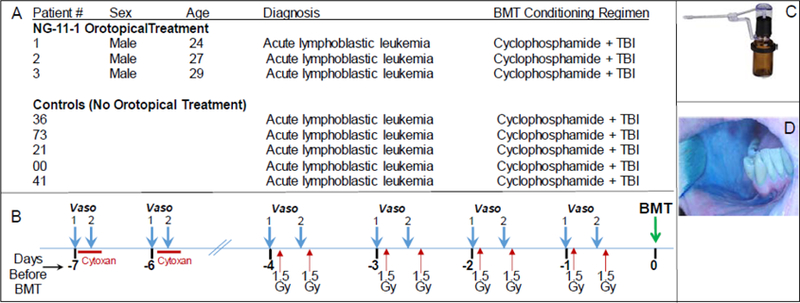
(A) Study patient parameters. (B) Schematic summary of the CY+TBI conditioning regimen, and the orotopical vasoconstrictor which was applied 20 min before and 25–30 min after the start of the cyclophosphamide IV administration; it was also applied 20 min before each of the 1.5 Gy total body irradiation doses. (C) Madomizer spray applicator used to apply vasoconstrictor; 30 × 0.10 ml sprays were applied to the patients’ oral cavities. (D) The orotopical NG-11–1 formulation contains a blue coloring to enable clinical staff to monitor coverage of oral surfaces during administration.

Baseline features of the three topical vasoconstrictor patients and the five control patients are shown in Figure 1, panel A. Briefly, treated patients were males aged 24, 27 and 29 years; each had been diagnosed with acute lymphoblastic leukemia, and each had failed at one previous course of chemotherapy. To qualify for enrollment, they had to be at least 18 years old, with no evidence of oral mucositis or active infection, no existent hypertension, and no abnormality in ECG. The five control patients also had been diagnosed with acute lymphoblastic leukemia, had been enrolled in the previous 12 months in the same UW Bone Marrow Transplant Program, and were of similar ages to the drug-treated patients.

The three topical vasoconstrictor-treated patients received a formulation containing 16.3 mM phenylephrine, an α-adrenergic vasoconstrictor formulated in an aqueous-alcohol vehicle with excipients that was specifically designed [18] for transmucosal, orotopical delivery of adrenergic vasoconstrictors. 3.0 ml of topical drug formulation was applied to patient’s mouths as 30 × 0.1 ml pulses from a Madomizer spray applicator (Figure 1C). For each patient, the study physician directed the 30 spray pulses preferentially to the buccal, lingual and sub-lingual surfaces on which OM lesions historically appeared. The formulation contains a blue coloring that enabled staff to direct and judge drug delivery and coverage (Figure 1D). Figure 1B shows: i) the standard administration schedule for cyclophosphamide (60 mg/kg/day x 2 days) and total body irradiation (1.5 Gy twice/ day × 4 days), as well as ii) the time points at which vasoconstrictor was applied. Orotopical vasoconstrictor was applied: i) 15–20 min before IV cyclophosphamide was initiated, ii) 25–30 min after IV cyclophosphamide was initiated, and iii) 15–20 min before every 1.5 Gy irradiations. No food or drink was consumed between orotopical drug application and irradiation. Blood pressure was monitored for 15 min after each orotopical drug dose, and no deviations were detected.

The sprayed and directed application of 3.0 ml of drug formulation was originally designed for use in pediatric patients, to avoid inadvertent swallowing of the drug. There were no enrolled children, and in future studies simple “swish and spit” administration will be used.

### Oral mucositis assessments

Oral mucositis severity was scored each day by a study physician using the WHO Mucositis Scale and the RTOG common toxicity criteria. Patients were evaluated using the Oral Mucositis Daily Questionnaire (OMDQ) and the Mouth Pain Categorical Rating Scale to assess oral pain. These assessments were performed for seven days prior to bone marrow transplant and continued until any evidence of OM had resolved or the patient was discharged from the hospital. After discharge from the hospital, assessments were repeated at the 1–2 week follow-up clinic visit and the three month follow-up clinic visit to be sure OM had resolved and no unexpected side effects were noted. Use of total parenteral nutrition as well as the ability to eat and drink were recorded. Doses of opioid and non-opioid analgesics were recorded.

## Discussion

The occurrence of oral mucositis during radiotherapy and chemotherapy is common. Clinical management of the pain and narcotic side effects, dehydration, dysphagia and weight loss, oral and septic infections, and substantial costs to HMOs are significant problems. The U.S. Cancer Pain Relief Committee concluded that “effective treatments to reduce the pain and functional impairment of oral mucositis are needed in this patient population [19].” There is currently no standard of care for either the prevention or treatment of the oral mucositis encountered in chemotherapy and radiotherapy treatment patients.

The use of oral cryotherapy to reduce OM severity [20] is in itself an acknowledgement of the oral vasoconstriction strategy, and the observed absence of tumor protection with oral cryotherapy [21] as measured by five-year tumor recurrence rates, supports oral mucosal vasoconstriction as a general protective strategy. An earlier report from our laboratory [13] also showed no radiation protection of three, histologically diverse, xenograft, tumor populations that received direct topical application of phenylephrine, epinephrine or norepinephrine 15 minutes before receiving tumoricidal irradiation. This outcome was consistent with the historical observation that human tumor vasculature lacks the smooth muscle cells with adrenergic receptors that are required to enable a response to an adrenergic vasoconstrictor.

An adrenergic vasoconstrictor, orotopically applied in a one minute “swish and spit” administration, provides several advantages over the oral cryotherapy approach, including: i) there is none of the discomfort associated with holding a mouth full of ice, ii) it can be used in head and neck radiotherapy patients where ice cannot, iii) the adrenergic agonist-induced vasoconstrictor effect can be controlled in a predictable manner through standard dose and application time strategies, i.e., a single vasoconstrictor application whose transient hypoxic effect dissipates quickly after a daily radiotherapy session, or, multiple applications over several hours to sustain the exclusion of blood-borne chemotherapy from the oral mucosa through 1–2 plasma half-lives of the systemic chemotherapy drug(s), iv) protection of the oral cavity and tongue surface epithelia that contact the orotopically administered vasoconstrictor could also confer protection to taste buds.

**Figure 2.**
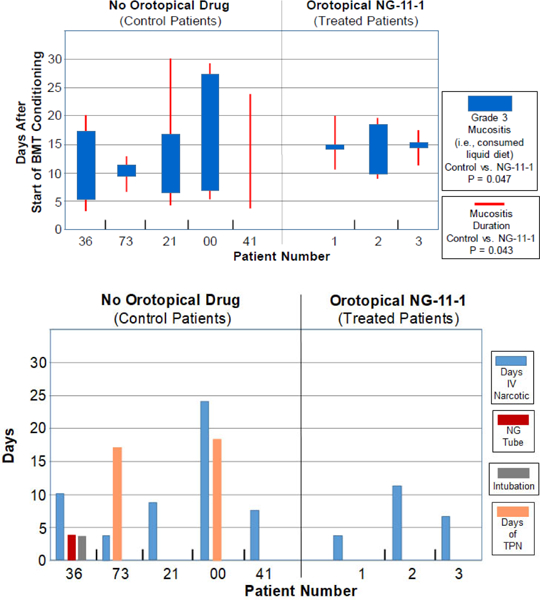
(A) Incidence and duration of Grade 3 Mucositis (i.e., consumption of liquid food) (blue bars) or Any Mucositis (red bars) in the days following initiation of the CY+TBI conditioning regimen in historical controls (Left panel) or topical vasoconstrictor-treated patients (Right panel). (B) Duration of supportive care steps taken in management of mucositis in historical controls and topical vasoconstrictor patients. NG Tube, nasogastric tube; TPN, total parenteral nutrition.

In this study, the physician photographed the patient’s oral cavity at every visit. Figure 2A shows the daily scores for both “any mucositis” (red lines), and for “Grade 3 mucositis” (blue bars). Irrespective of the images of the oral cavity on a given day, a “Grade 3” OM score was assigned by the physician on any day in which the patient by their choice consumed only “liquid” food. The means and std. devs. for days of “Grade 3 oral mucositis duration” and “any mucositis duration” in vasoconstrictor-treated patients were compared to the scored values in the five control patients. We were encouraged to see that in both cases the P values were below 0.05, but nonetheless, this is still a report of three patients in this pilot case report. Future studies will: i) include larger patient populations and ii) test additional, higher, vasoconstrictor doses.

Figure 2B shows that though either nasogastric feeding or total parenteral nutrition was required in 3 of the 5 CY+TBI control patients, none of the three vasoconstrictor-treated patients required this intervention.

**Figure 3.**
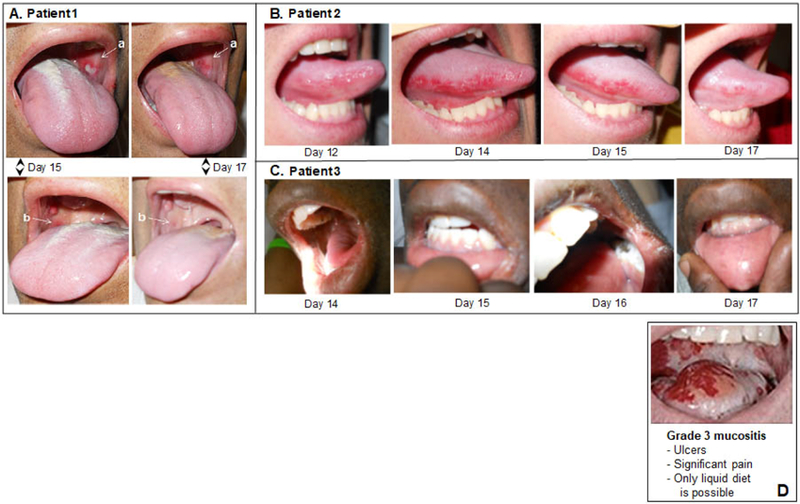
Representative oral images on the indicated days following initiation of CY+TBI conditioning regimen in topical vasoconstrictor-treated patients. (A) Patient 1, (B) Patient 2, (C) Patient 3. (D) Example of Grade 3 mucositis as scored using WHO oral mucositis criteria.

Figure 3 panels A, B, C, show sample images from the three vasoconstrictor-treated patients taken on the 3–4 worst OM days, typically 13–16 days after initiation of the CY+TBI regimen. The images illustrate and corroborate both the assigned scores and the peak OM days when compared to the scores assigned in Figure 2A. In these three vasoconstrictor-treated patients, the OM severity is: i) modest in intensity and ii) rapidly resolved, i.e., compare resolution of the a and b lesions in Patient 1 between Days 15 and 17, and the onset and resolution of the inflamed tongue lesions in Patient 2. Figure 3, panel D shows a Grade 3 OM image that has been commonly associated with the World Health Organization scoring criteria for OM. Though the three vasoconstrictor-treated patients technically met the WHO Grade 3 OM “score” because they chose liquid-only diets on the indicated days shown in Figure 2A, the severity of inflammation and tissue degradation in the vasoconstrictor patients was far less severe than that seen in the standard WHO Grade 3 image.

## Conclusion

The orotopically applied NG11–1 vasoconstrictor formulation of phenylephrine was shown to substantially suppress the incidence and severity of both the “Grade 3” and “any” oral mucositis when compared to matched control patients, all of whom received the same CY+TBI conditioning regimen within the same UW Bone Marrow Transplant Program. The liquid orotopical formulation was easily tolerated by patients both in its ease of use and lack of side effects.
